# *Ceiba speciosa* (A. St.-Hil.) Seeds Oil: Fatty Acids Profiling by GC-MS and NMR and Bioactivity

**DOI:** 10.3390/molecules25051037

**Published:** 2020-02-25

**Authors:** Sergio Rosselli, Rosa Tundis, Maurizio Bruno, Mariarosaria Leporini, Tiziana Falco, Rossella Gagliano Candela, Natale Badalamenti, Monica R. Loizzo

**Affiliations:** 1 Department of Agricultural, Food and Forest Sciences (SAAF), Università degli Studi di Palermo. Viale delle Scienze, ed. 4, I-90128 Palermo, Italy; 2 Centro Interdipartimentale di Ricerca “Riutilizzo bio-based degli scarti da matrici agroalimentari” (RIVIVE), Università di Palermo, 90128 Palermo, Italy; 3 Department of Pharmacy, Health and Nutritional Sciences, University of Calabria, 87036 Rende (CS), Italy; rosa.tundis@unical.it (R.T.); mariarosarialeporini@tiscali.it (M.L.); tiziana.falco@unical.it (T.F.); monica_rosa.loizzo@unical.it (M.R.L.); 4 Department of Biological, Chemical and Pharmaceutical Sciences and Technologies (STEBICEF), Università degli Studi di Palermo. Viale delle Scienze, ed. 17, I-90128 Palermo, Italy; r.gaglianocandela88@gmail.com (R.G.C.); natale92@live.it (N.B.)

**Keywords:** fixed oil, GC-MS, NMR, antioxidant, diabetes, obesity

## Abstract

This study aimed to evaluate the chemical composition by gas chromatography-mass spectrometry (GC-MS) and Nuclear Magnetic Resonance (NMR) analyses, the antioxidant activities evaluated by different in vitro assays namely 2,2-diphenyl-1-picrylhydrazyl (DPPH), 2,2′-azino-bis(3-ethylbenzothiazoline-6-sulphonic acid) (ABTS), Ferric Reducing Ability Power (FRAP), and β-carotene bleaching tests, and the inhibitory effects of enzymes linked to obesity (lipase, α-amylase, and α-glucosidase) of fixed seed oil of *Ceiba speciosa* (A. St.-Hil.). Fourteen compounds were identified. Linoleic acid (28.22%) was the most abundant followed by palmitic acid (19.56%). Malvalic acid (16.15%), sterculic acid (11.11%), and dihydrosterculic acid (2.74%) were also detected. *C. speciosa* fixed oil exerted a promising ABTS radicals scavenging activity with an IC_50_ value of 10.21 µg/mL, whereas an IC_50_ of 77.44 µg/mL against DPPH^+^ radicals was found. *C. speciosa* fixed oil inhibited lipase with an IC_50_ value of 127.57 µg/mL. The present investigation confirmed the functional properties of *C. speciosa* fixed oil, and proposes its use as valuable source of bioactive constituents.

## 1. Introduction

Metabolic syndrome is a multiple factor syndrome in which there is a co-existence of several risk factors such as hyperglycaemia, dyslipidaemia, and hypertension. This syndrome is frequently linked to obesity [1]. In fact, a dysregulated production of adipokines by adipocytes contributes to insulin resistance and atherosclerosis with an increase risk in the development of thrombosis [2,3]. Epidemiological studies evidence that worldwide the number of obese people is approximately 2.1 billion. This number continues to rise. Moreover, it is estimated that obesity and linked pathology kill over 3 million people each year [4]. Oxidative stress plays critical roles in the pathogenesis of both diabetes and obesity. In diabetic patients, oxidative stress impairs glucose uptake in both muscle and fat tissue, as well as reduces insulin secretion by *β* cells [5].

The recovery of plant species useful in the prevention and treatment of this syndrome is a research topic of great interest. In this context, data obtained by ethnobotany investigation could propose the use of wild vegetables, growing spontaneously as part of the diet, since they represent a possible new trend in contemporary nutrition due to their health benefits [6].

Lipase, α-amylase, and α-glucosidase represent key enzymes involved in the hydrolysis of fat and dietary carbohydrates in humans [7].

*Ceiba speciosa* (A. St.-Hil.) (Floss Silk Tree), known worldwide with the synonym *Chorisia speciosa* A. St.-Hil. and once assigned to the family Bombacaceae, is now included within Malvaceae [8]. It is a 10–20 m tree with pale green lobed leaves and beautiful pink flowers. The young trees grow straight and narrow and then the trunk becomes bottle-shaped as it ages, covered with big, triangular spines. The fruits are pear-shaped capsules filled with many pea-size seeds embedded in silky white floss. On ripening, the capsules open to expose masses of this white silky, cottony material that falls to ground. The silk of the pod (the so-called “false kapok) is used as waterproof stuffing in pillows, cushions, and mattresses. Thin strips of the bark have been used to make rope. The wood is light and soft and is used for the manufacture of match sticks. It is native to the tropical and subtropical forests of South America. It has several local common names, such as “palo borracho” (in Spanish), samu′ũ (in Guarani), paineira (in Brazilian Portuguese), and “toborochi” in Bolivia, meaning “tree of refuge” or “sheltering tree”. 

In the pre-Columbian Mesoamerican cultures, particularly among the Mayan people, the tree played a significant role in ecology, culture, and rituals since it was supposed to connect the underworld, the terrestrial realm, and the sky [9]. With regard to European territory, it is naturalized on Madeira Island. Consequently, the nuclei present in the urban area of Palermo city represent the second case of casual naturalization for the whole Europe and the first for the Mediterranean area [10].

According to Lojacono-Pojero [11], its seeds were presumably planted in a private garden of the city of Palermo around 1896 by a “lady” coming from Brazil and then in the Colonial Garden of Palermo in order to assess the quality and amount of available fibres—the so-called “false kapok”—which could be extracted from their fruits and transformed for textile purposes. Successively, its seeds were tested for its technical properties in order to overcome the lack of fuel during the embargo suffered by Italy under the fascist regime [12]. Plants from *Chorisia* genus are traditionally used for the treatment/prevention of diabetes, obesity, hypertension, diarrhoea, peptic ulcer, asthma, rheumatism, and infections [13]. 

Previous phytochemical investigations showed the occurrence of flavonoids in the flowers [14] and leaves [15,16], anthocyanes in the flowers [17], steroids [16,18] and fatty acids (malvalic acid, sterculic acid) in the seeds [18,19], monosaccharides in the fruits [20], in the gum [21] and seeds [22], heteropolysaccharides in the floss silk [23], in the mucilage of flowers [14], and in the mucilage of leaves [24]. 

Recently, β-amyrin and verbascoside, firstly reported in the family, and other known compounds, including the flavonoids rhoifolin and tiliroside, were isolated from the alcoholic extract of leaves of *C. speciosa* cultivated in Egypt [25]. The presence of these two flavonoid glycosides, rhoifolin and tiliroside [25], having noteworthy anti-inflammatory, antioxidant, anti-hypertensive, hepatoprotective, selective cytotoxic antidiabetic, anti-hyperlipidemic and antimicrobial effects, induced the authors to produce them from the callus of *C. speciosa*. [26]. Furthermore, the leaf extracts showed good antioxidant, anti-inflammatory, antipyretic, antibacterial, and antifungal activities [25,27]. However, few studies have investigated the chemical composition of its fixed oil [19,28] and neither investigated its bioactivity. 

For this purpose, *Ceiba speciosa* seeds fixed oil was analysed for its chemical composition, antioxidant activity by using different in vitro assay namely 2,2-diphenyl-1-picrylhydrazyl (DPPH), 2,2′-azino-bis-3-ethylbenzothiazoline-6-sulfonic acid (ABTS), Ferric Reducing Antioxidant Power (FRAP), and hypolipidaemic and hypoglycaemic activities through the inhibition of pancreatic lipase, α-amylase and α-glucosidase.

## 2. Results and Discussion

### 2.1. Chemical Profiling

**Figure 1:**
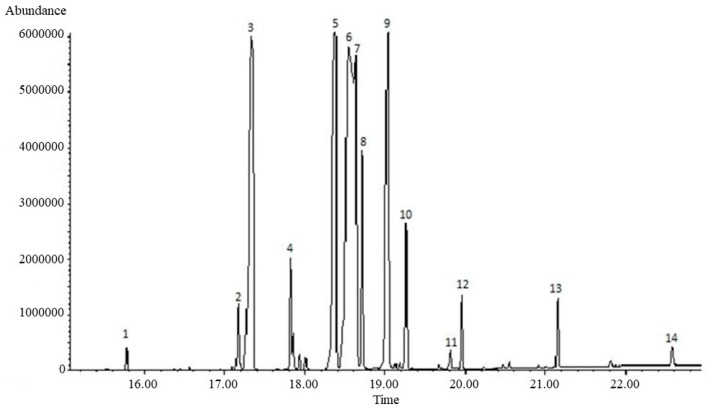
Identified fatty acids. 1: Myristic acid; 2: Palmitoleic acid; 3: Palmitic acid; 4: Margaric acid; 5: Malvalic acid; 6: Linoleic acid; 7: Oleic acid; 8: Stearic acid; 9: Sterculic acid; 10: Dihydrosterculic acid; 11: Gondoic acid; 12: Arachidic acid; 13: Behenic acid; 14: Lignoceric acid.

Table 1 reports the main fatty acids identified by GC-MS analysis in *C. speciosa* seeds n-hexane extract. A total of 14 main compounds were identified (Figure 1). Linoleic acid (28.22%) was the most abundant fatty acid, followed by palmitic acid (19.56%). The three cyclopropane fatty acids, identified by comparison with published mass spectra [29] such as malvalic acid, sterculic acid and dihydrosterculic acid accounted for 16.15, 11.11, and 2.74%, respectively. Bohannon and Kleiman [28] reported percentage of malvalic acid and sterculic acid of 12.4 and 10.0%, respectively.

Previously, lower percentages of these cyclopropane fatty acids (9.0 and 6.0% for malvalic and sterculic acids, respectively) were found by Petronici et al. [19]. The chemical analysis of *Ceiba* pentandra seed oil from Malaysia showed the presence of linoleic acid (38.82%), palmitic acid (24.31%), and oleic acid (21.88%) as the most abundant fatty acids [30]. Malvalic and sterculic acids were also identified with percentage of 7.18 and 2.96%, respectively [30]. Differently, Kiran and Rao [31] reported a lower percentage of linoleic acid for the seed oil of *C. pentadra* from India.

The GC-MS analysis of the trans-methylated fixed oil showed the occurrence of eleven fatty acids methyl esters and three other compounds that could not be identified through our library.

**Figure 2:**
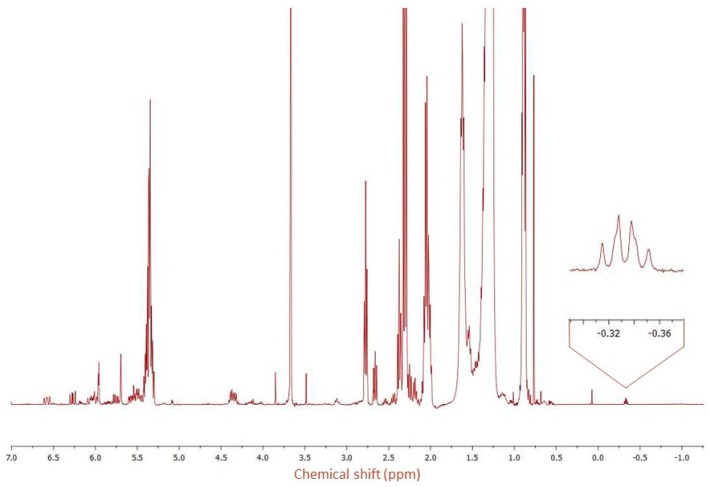
^1^H-NMR spectrum of fixed oil trans-methylation (400 MHz, CDCl_3_) in the region −1.5 to 7.0 ppm. Expansions of the peaks at −0.37 to −0.29 ppm is also shown.

Therefore, on the basis of previous works carried out on the genus *Ceiba* [28,30], which revealed the presence of cyclopropanic fatty acids, 1D and 2D-NMR techniques were employed to search for the characteristic signals of these acids. Figure 2 shows signals, in the ^1^H-NMR spectrum, characteristic of dihydrosterculic, sterculic, and malvalic methyl ester. The signal between −0.33 (q) can be attributed to one proton of the methylene moiety in the cyclopropanic ring of methyl dihydrosterculate [32]; while the singlet at 0.77 ppm is generated by cyclopropenic methylene protons, such as those present on sterculic and malvalic methyl esters [33,34].

**Figure 3:**
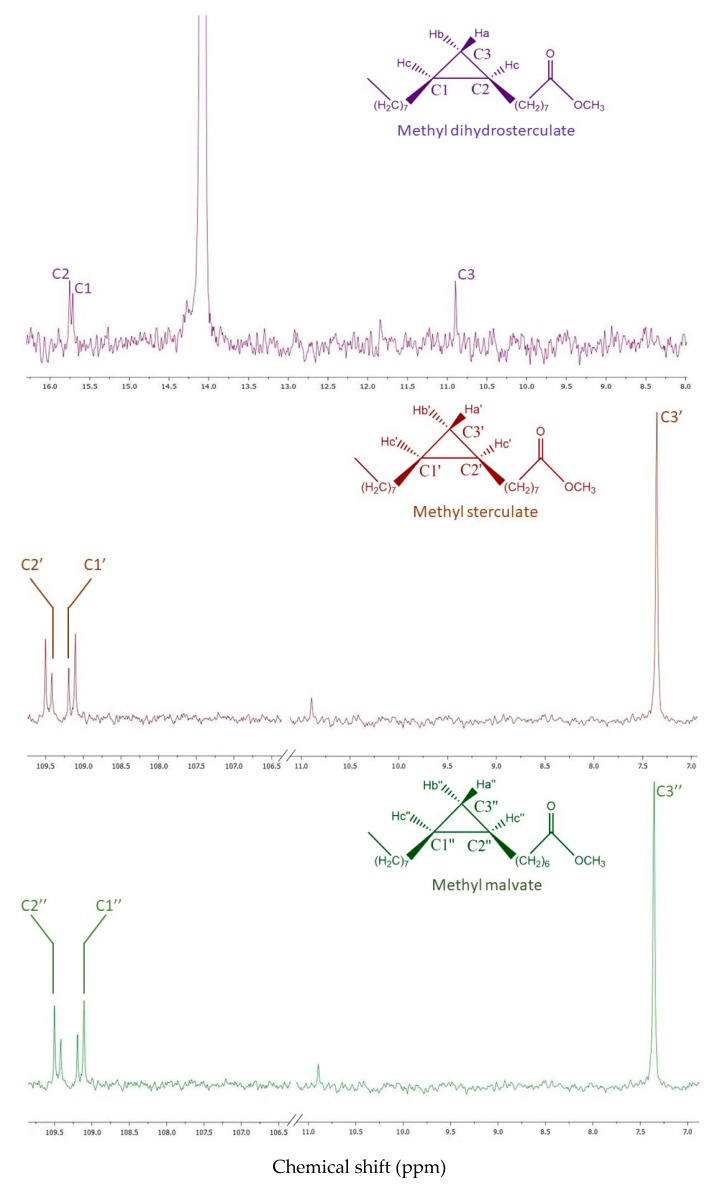
^13^C-NMR spectrum (100 MHz, CDCl_3_) of methyl dihydrosterculate, methyl sterculate, and methyl malvate.

**Figure 4:**
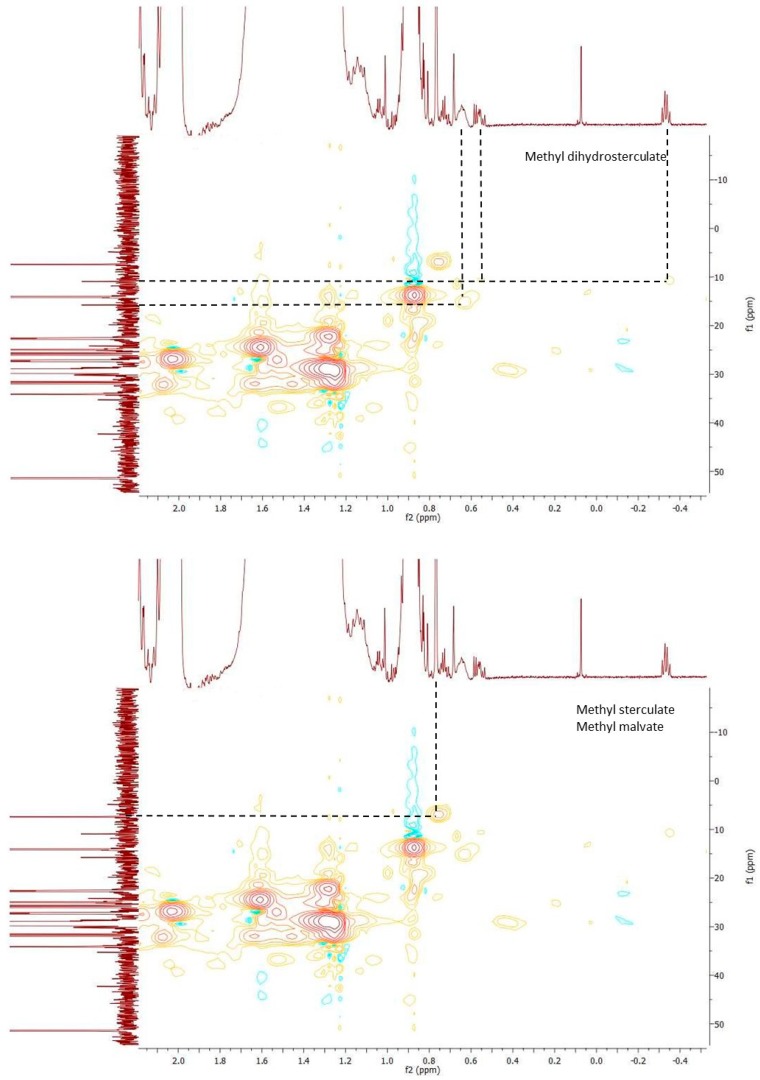
HSQC spectrum showing correlations in methyl dihydrosterculate, methyl sterculate, and methyl malvate.

However, due to the overlapping of signals in the ^1^H-NMR spectrum, ^13^C-NMR spectrum and heteronuclear correlation (HSQC) (Figure 3 and Figure 4) were registered in order to confirm the presence of the cyclopropanoid acids. The ^13^C NMR spectrum showed 3 typical carbon signals at 10.89 ppm for the methylene and at 15.71 and 15.75 ppm for the methine carbons of the cyclopropane ring, respectively, of methyl dihydrosterculate. The methylene protons (δ_Ha_ = 0.57, δ_Hb_ = −0.34) showed HSQC correlation with a secondary carbon C3 at δ_C3_ = 10.89; a single cross correlation peak was also visible for the methine protons (δ_Hc_ = 0.63) and the carbons C1 and C2 at 15.71 and at 15.75 ppm, respectively. These data strongly suggested the occurrence of methyl dihydrosterculate. In turn, for the sterculic and malvalic methyl ester, the peak of the cyclopropenic methylene protons at 0.77 ppm correlates with ^13^C NMR signal at 7.35 ppm (C-3).

Furthermore, the signals at 109.19 ppm and 109.42 ppm, and 109.11 ppm and 109.50 ppm were in perfect agreement [34] with those reported for sterculic and malvalic methyl ester, respectively. Based on these considerations, the mass-spectra and the retention indexes of the three GC unidentified compounds were searched in the literature [29] and a perfect match with the cyclopropanoid fatty acid methyl esters was found.

### 2.2. Antioxidant Activity

The antioxidant activities of *C. speciosa* seed fixed oil were investigated by using two radical scavenging assays, namely DPPH and ABTS. The protection of lipid peroxidation (β-carotene bleaching test) as well as the effect on iron, one of the main involved ion in the oxidation process, was studied. Data are reported in Table 2.

Fixed oil showed a concentration-dependent antioxidant activity in both radical scavenging test. In particular, *C. speciosa* fixed oil exerted a promising ABTS radicals scavenging activity with an IC_50_ value of 10.21 µg/mL, whereas it had an IC_50_ of 77.44 µg/mL against DPPH^+^ radical. These results may be justified taking into consideration the different mechanisms of action of the two radicals scavenging assays.

A lower effect was recorded in the FRAP test in which the FRAP value was 3-times lower than that the positive control BHT. Percentage of 37.36 and 36.98% were obtained in β-carotene bleaching test at oil concentration of 100 µg/mL after 30 and 60 min of incubations, respectively.

To our knowledge, this is the first report on the antioxidant activity of fixed oil. Previously, Dörr et al. [35] revealed that *C. speciosa* aqueous steam bark extract exerted a promising DPPH radical scavenging potential. A promising DPPH radical scavenging effect was observed, also by Krüger Cardoso Malheiros et al. [36], who found percentage of 85.13 and 88.95% at concentration of 50 μg/mL for raw aqueous bark extract and ethanol extract, respectively. Both extracts are rich in kaempferol, cholorogenic acid and caffeic acid. Different extracts from *C. speciosa* leaves and stem were studied for their antioxidant activity [25]. Generally, the stem was a little more active than leaves and fruits with percentage of inhibition of 81.2, 78.87, and 77.5% for stem, leaves, and fruits ethanol extract, respectively. More recently, Refaat et al. [27] compared the DPPH radicals scavenging ability of different extracts from *C. speciosa* and *C. chodatii*. Ethyl acetate extracts had the most active fractions followed by aqueous extract. Comparing different investigated parts, the following trend: flowers > fruits > leaves > seeds was found.

### 2.3. Inhibition of Enzymes Linked to Obesity

The search for new agents useful for the prevention of obesity and related pathologies is a topic of great interest for the scientific community. *C. speciosa* fixed oil was investigated for its potential anti-obesity and related disorder prevention effect. Data are reported in Table 3. A concentration-dependent manner should be observed for all investigated samples independently by the enzyme assays used. IC_50_ values of 135.69 and 158.22 µg/mL were recorded against α-amylase and α-glucosidase, respectively, whereas IC_50_ value of 127.57 µg/mL was found against lipase enzyme. The in vivo anti-hyperglycaemic effect of *C. insignis* was observed by El-Alfy et al. [37]. Dried leaves’ total and aqueous extracts as well as ethyl acetate fraction administered intraperitoneally (150 mg/kg) exerted a promising anti-hyperglycaemic by decreasing blood glucose level effect in alloxan-induced diabetic rats as compared with the positive control metformin.

The determination of the median lethal dose (LD_50_) of the total and the aqueous extracts and the successive fractions revealed that this plant is safe. The effect of *C. pentandra* on obese wistar albino rats was observed by Patil et al. [38]. Oral administration of 125 mg/kg of leaves total ethanol extract prevented the increase of body weight, as demonstrated by the reduction of body max index (0.16 g/cm^2^) as compared to the cafeteria control group without any effect on appetite. Moreover, administration of *C. pentandra* extract did not decrease total cholesterol content, LDL or HDL levels. Therefore, it is possible that its action is mediated by preventing the breakdown of dietary fat in the gastrointestinal tract. The acute toxicity study revealed that administration of *C. pentandra* extract was safe up to 5000 mg/kg. More recently, Refaat et al. [27] demonstrated that both *C. speciosa* and *C. chodati* total ethanol extracts of different plant portions at 5 µg/ mL reduced the lipid droplets in 3T3-L1 preadipocytes. However, at higher doses, an induction on adipogenesis was observed.

## 3. Materials and Methods

### 3.1. Chemicals and Reagents

*n*-Hexane and methanol were from Honeywell (Seelze, Germany). Tween 20, Fatty Acid Methyl Ester (FAME) Standards, BF_3_/MeOH 12% solution, ascorbic acid, Folin-Ciocalteu reagent, sodium carbonate, potassium hydroxide, sodium hydroxide, butylated hydroxytoluene (BHT), propyl gallate, 2,2-diphenyl-1-picrylhydrazyl (DPPH), tripyridyltriazine (TPTZ), 2,2′-azino-bis(3-ethylbenzothiazoline-6-sulfonic) acid (ABTS) solution, β-carotene, linoleic acid, Orlistat, Trizma base, 4-nitrophenyl octanoate (NPC), maltose, α-amylase and lipase from porcine pancreas, α-glucosidase from *Saccharomyces cerevisiae*, *O*-dianisidine dihydrochloride, and PGO enzyme preparation were purchased from Sigma-Aldrich S.p.a. (Milan, Italy). Acarbose from Actinoplanes sp. was obtained from Serva (Heidelberg, Germany).

### 3.2. Plant Materials

The fruits of *C. speciosa* were harvested in May 2019 from different plants growing in the campus of Palermo University, Parco d’Orleans, Palermo (Sicily, Italy) (38°06′10″ N, 13°20′52″ E) at 47 m. a.s.l. In particular, the gathering of fruits was carried out on the campus by collecting them randomly from 20 healthy plants. The collected fruits were placed in a laboratory and air dried for about a month. The seeds were manually drawn out from the fruits and used for the analysis. The flowers of *C. speciosa* were collected in November 2019 from different plants growing on the campus of Palermo University, Parco d’Orleans, Palermo (Sicily, Italy) (38°06′10″ N, 13°20′52″ E) at 47 m. a.s.l. The vouchers are deposited at the Herbarium Mediterraneum Panormitanum (PAL).

### 3.3. Extraction Procedure

Seeds (50 g) were removed from the fruits, then reduced to a fine powder using a blender type A11 basic and extracted twice with *n*-hexane (400 mL) under stirring at room temperature for 72 h. The solvent was evaporated at 40 °C using a Buchi rotavapor R-200 (Germany) to give 7.33 g of oil (yield 14.6%) [39]. The resulting oil was kept away from light and at low temperature.

### 3.4. Gas Chromatography—Mass Spectrometry (GC-MS) Analyses

Fixed oil (100 mg) was subjected to basic transmethylation using potassium hydroxide in methanol. To a solution of the oil (100 mg in 2 mL of hexane), 0.2 mL of 2 M methanolic KOH was added and allowed to stir for 2 min or at 30 °C [40]. An aliquot of the upper hexane layer was directly taken and analysed by gas chromatography associated with mass spectrometry (GC-MS), using a Hewlett-Packard 6890 gas chromatograph (Agilent, Milan, Italy) equipped with a non-polar HP-5 capillary column (30 m × 0.25 mm, 0.25 μm), associated with a Hewlett-Packard 5973 mass spectrometer completed by Hewlett Packard Chemstation data system (Agilent, Milan, Italy). The ionization of the sample constituents was performed in electronic impact (EI, 70 eV). The analyses were carried out by using the following temperature schedule: isotherm at 50 °C for 5 min, temperature increase from 60 to 250 °C of 14 °C/min, and finally isotherm at 250 °C for 10 min. Helium is used as a carrier gas. Compounds identification corresponding to methyl esters (FAMEs) was based on the comparison of the mass spectral data with the Wiley 128 library and referring to the spectral data of a standard mixture of FAMEs. The compounds’ relative concentrations were calculated based on peak areas without using correction factors.

### 3.5. Nuclear Magnetic Resonance (NMR) Analyses

NMR spectra were acquired with CDCl_3_ as solvent on a Brucker Avance II spectrometer, at Centro Grandi Apparecchiature (Palermo, Italy), operating at 400 MHz (^1^H) or 100 MHz (^13^C) frequencies. The chemical shifts are reported relative to the chloroform residual peak (7.27 ppm for ^1^H NMR).

### 3.6. Radical Scavenging Activity Assays

The 2,2-diphenyl-1-picrylhydrazyl (DPPH) radicals scavenging activity was performed as previously reported [41]. Absorbance modifications as a consequence of DPPH radical reaction was measured at 517 nm. 2,2′-Azino-bis(3-ethylbenzothiazoline-6-sulphonic acid) (ABTS) radical scavenging ability was measured following the protocol previously described [42]. ABTS scavenging activity (%) was calculated as follows: [(A′– A)/A′] × 100, where A′ is the absorbance of the control reaction and A is the absorbance in the presence of the extract. Ascorbic acid was employed as a positive control in both assays.

### 3.7. β-Carotene Bleaching Test

The *β*-Carotene Bleaching Test measured the ability of the extract to protect lipid substrate from peroxidation. The test was applied following the procedure described by Loizzo et al. [41]. In brief, extract at different concentrations, β-carotene solution, linoleic acid and 100% Tween 20 were mixed. The absorbance was measured at 470 nm against a blank at t = 0 and after 30 and 60 min of incubation. Propyl gallate was used as a positive control.

### 3.8. Ferric Reducing Ability Power (FRAP) Assay

The FRAP assay was done following the previously described procedure [43]. The FRAP value represents the *ratio* between the slope of the linear plot for reducing Fe^3+^-TPTZ reagent by different extract compared to the slope of the plot for FeSO_4_.

### 3.9. Pancreatic Lipase Inhibitory Activity

The pancreatic lipase inhibitory activity was investigated following the procedure of El-shiekh with some modifications [44]. The enzyme at concentration of 1 mg/mL was measured using 4-nitrophenyl octanoate as a substrate, 5 mmol/L in dimethylsulfoxide solution. Extracts at different concentrations were left to react with enzyme, substrate, and Tris-HCl buffer (pH 8.5) at 37 °C for 30 min. The absorbance was measured (405 nm). Orlistat was used as a positive control.

### 3.10. Carbohydrates-Hydrolysing Enzymes Inhibitory Activity

The carbohydrates-hydrolysing enzymes’ inhibitory activity was investigated using α-amylase and α-glucosidase as enzymes. The procedure is previously described [41]. Results are expressed as IC_50_ values and acarbose was used as positive control in both assays.

### 3.11. Statistical Analysis

The concentration-response curve and the inhibitory concentration 50% (IC_50_) was calculated by using Prism GraphPad Prism version 4.0 for Windows, GraphPad Software (San Diego, CA, USA). The same software was used to perform one-way ANOVA followed by a multicomparison Dunnett’s test (α = 0.05): **** *p* < 0.0001, *** *p* < 0.001 compared with the positive controls.

## 4. Conclusions

The present study assessed the chemical profile, α-amylase, α-glucosidase, and lipase inhibitory activity, and the antioxidant properties of *C. speciosa* fixed oil obtained from plant domesticated in Palermo (Sicily). Linoleic acid was the most abundant fatty acid followed by palmitic acid. Three cyclopropane fatty acids, namely malvalic, sterculic, and dihydrosterculic, are also identified and their concentration is in line with those reported for other *Ceiba* species. The fixed oil exerts both hypoglycaemic and anti-obesity effects and for this reason data obtained from this research activity could help to support the use of the edible oil of a local plant due to its antioxidant properties and for the prevention of obesity and related disorders.

## Figures and Tables

**Table 1:** The main fatty acids of *C. speciosa* seeds n-hexane extract.

Peak	R_t_ (min)	Fatty acid ^a^	%
1	15.78	Myristic acid	0.38 ± 0.01
2	17.16	Palmitoleic acid	1.42 ± 0.24
3	17.33	Palmitic acid	19.56 ± 1.32
4	17.81	Margaric acid	1.41 ± 0.02
5	18.37	Malvalic acid ^b^	16.15 ± 2.14
6	18.54	Linoleic acid	28.22 ± 2.56
7	18.63	Oleic acid	8.93 ± 0.21
8	18.71	Stearic acid	4.33 ± 0.14
9	19.03	Sterculic acid ^b^	11.11 ± 2.88
10	19.26	Dihydrosterculic acid ^b^	2.74 ± 0.16
11	19.81	Gondoic acid	0.40 ± 0.03
12	19.95	Arachidic acid	1.27 ± 0.13
13	21.14	Behenic acid	1.35 ± 0.21
14	22.58	Lignoceric acid	0.52 ± 0.03
		Total identified	97.79

Data are reported as mean ± standard deviation (*n*= 3). ^a^ Identified al methyl esters ^b^ Identification by comparison with published data [29].

**Table 2:** Antioxidant activity of *C. speciosa* fixed oil.

	DPPH IC_50_ (µg/mL)	ABTS IC_50_ (µg/mL)	FRAP μMFe (II)/g	β-carotene Bleaching Test t = 30 Min % at 100 μg/mL	β-carotene Bleaching Test t = 60 Min % at 100 μg/mL
*Chorisia speciosa*	77.44 ± 2.93 ****	10.21 ± 2.35 **	21.12 ± 2.14 ****	37.36 ± 2.05 ****	36.98 ± 2.11 ****
Positive control					
Ascorbic acid	5.04 ± 0.84	1.76 ± 0.06			
BHT			63.20 ± 2.34		
Propyl gallate				0.09 ± 0.04	0.09 ± 0.04

Data are expressed as means ± S.D. (*n* = 3). Differences within and between groups were evaluated by one-way ANOVA followed by a multi-comparison Dunnett’s test α = 0.05): **** *p*< 0.0001, ** *p* < 0.01, compared with the positive controls.

**Table 3:** *C. speciosa* fixed oil anti-obesity activity IC_50_ (µg/mL).

Sample	α-Amylase	α-Glucosidase	Lipase
*C. speciosa*	158.22 ± 2.89 ****	135.69 ± 2.68 ****	127.57 ± 2.98 ****
Positive control			
Acarbose	50.01 ± 0.92	35.52 ± 1.23	
Orlistat			37.63 ± 1.01

Data are expressed as means ± S.D. (*n* = 3). Acarbose used as positive control in α-amylase and α-glucosidase tests. Orlistat used as positive control in the lipase test. Differences within and between groups were evaluated by one-way ANOVA, followed by a multicomparison Dunnett’s test (α = 0.05): **** *p* < 0.0001 compared with the positive control.
